# A Comparison Study Between CNN-Based Deformed Planning CT and CycleGAN-Based Synthetic CT Methods for Improving iCBCT Image Quality

**DOI:** 10.3389/fonc.2022.896795

**Published:** 2022-05-30

**Authors:** Bo Yang, Yankui Chang, Yongguang Liang, Zhiqun Wang, Xi Pei, Xie George Xu, Jie Qiu

**Affiliations:** ^1^ Department of Radiation Oncology, Chinese Academy of Medical Sciences, Peking Union Medical College Hospital, Beijing, China; ^2^ School of Nuclear Science and Technology, University of Science and Technology of China, Hefei, China; ^3^ Technology Development Department, Anhui Wisdom Technology Co., Ltd., Hefei, China; ^4^ Department of Radiation Oncology, First Affiliated Hospital of University of Science and Technology of China, Hefei, China

**Keywords:** iCBCT, registration, sCT generation, pelvic, CycleGAN

## Abstract

**Purpose:**

The aim of this study is to compare two methods for improving the image quality of the Varian Halcyon cone-beam CT (iCBCT) system through the deformed planning CT (dpCT) based on the convolutional neural network (CNN) and the synthetic CT (sCT) generation based on the cycle-consistent generative adversarial network (CycleGAN).

**Methods:**

A total of 190 paired pelvic CT and iCBCT image datasets were included in the study, out of which 150 were used for model training and the remaining 40 were used for model testing. For the registration network, we proposed a 3D multi-stage registration network (MSnet) to deform planning CT images to agree with iCBCT images, and the contours from CT images were propagated to the corresponding iCBCT images through a deformation matrix. The overlap between the deformed contours (dpCT) and the fixed contours (iCBCT) was calculated for purposes of evaluating the registration accuracy. For the sCT generation, we trained the 2D CycleGAN using the deformation-registered CT-iCBCT slicers and generated the sCT with corresponding iCBCT image data. Then, on sCT images, physicians re-delineated the contours that were compared with contours of manually delineated iCBCT images. The organs for contour comparison included the bladder, spinal cord, femoral head left, femoral head right, and bone marrow. The dice similarity coefficient (DSC) was used to evaluate the accuracy of registration and the accuracy of sCT generation.

**Results:**

The DSC values of the registration and sCT generation were found to be 0.769 and 0.884 for the bladder (*p* < 0.05), 0.765 and 0.850 for the spinal cord (*p* < 0.05), 0.918 and 0.923 for the femoral head left (*p* > 0.05), 0.916 and 0.921 for the femoral head right (*p* > 0.05), and 0.878 and 0.916 for the bone marrow (*p* < 0.05), respectively. When the bladder volume difference in planning CT and iCBCT scans was more than double, the accuracy of sCT generation was significantly better than that of registration (DSC of bladder: 0.859 vs. 0.596, *p* < 0.05).

**Conclusion:**

The registration and sCT generation could both improve the iCBCT image quality effectively, and the sCT generation could achieve higher accuracy when the difference in planning CT and iCBCT was large.

## Introduction

Cervical cancer is an important factor that endangers women’s lives (1), and radiotherapy is one of the main ways to treat cervical cancer. The most widely used radiotherapy techniques in clinical practice are IMRT (intensity modulated radiotherapy) (2) and VMAT (volumetric modulated radiotherapy) (3, 4), both of which can provide a high dose to the target area while protecting more organs at risk (OARs). Higher conformity requires higher accuracy of the patient’s position during treatment; thus, image-guided radiotherapy (IGRT) is used to monitor changes in the patient’s position and anatomical structure during clinical treatment. The acquisition of CT image again may increase the treatment burden and radiation, and CBCT image guidance is most widely accepted in clinical practice. However, the quality of CBCT images is poor due to the scattering and artifacts, which is typically not enough for dose calculation and adaptive radiotherapy. The iterative cone beam CT (iCBCT) combines the statistical reconstruction and Acuros CTS scattering correction algorithm (5, 6), which can achieve uniform imaging with less noise and higher quality. Nevertheless, the artifacts (cavity artifacts, etc.) still exist, which need to be improved.

In recent years, deep learning-based image processing methods have been widely applied to the field of medical imaging, including medical image segmentation (7–9), disease diagnosis (10, 11), medical image denoising (12), and medical image translation (13, 14). The development of deep learning technology has accelerated the process of clinical treatment and improved the mining of medical image information. For the inaccuracy of CBCT images, many scholars have made a lot of contributions to improve the quality of CBCT images based on deep learning methods; some of them used the planning CT (pCT) to be registered to the CBCT to obtain deformed planning CT (dpCT), which was used to approximately replace CBCT as the current treatment images. Duan et al. (15) proposed a patch-wise CT-CBCT registration unsupervised model for thoracic patients; Han et al. (16) used a segmentation similarity loss, in addition to the image similarity loss, to train the network to predict the transformation between the pancreatic CT and CBCT images. Liang et al. (17) developed a deep unsupervised learning (DUL) framework based on a regional deformable model for automated prostate contour propagation from pCT to CBCT. In addition, some scholars tried to generate sCT from CBCT images, which was used to replace CBCT as the current treatment images. Zhao et al. (18) used the modified CycleGAN to generate sCT from MV CBCT; the auto-segmentation and dose calculation based on sCT showed promising results. Liang et al. (19) compared the CycleGAN model with other unsupervised learning methods and demonstrated that CycleGAN (20) outperformed the other models on sCT generation. Chen et al. (21) retrained the head model in the pelvic region, and the improvement of the accuracy proved the generalization feasibility of sCT generation.

However, the registration accuracy of CT-CBCT depends more on the consistency of pCT and CBCT images. Deformable image registration (DIR) enabled accurate contour propagation and dose calculation for head and neck (22), but obtained lower accuracy in more complex anatomical regions such as the lung (23) and pelvis (24). Due to the daily deformation of the patient’s anatomy, especially for cases with large differences in bladder volumes in cervical cancer patients, the accuracy of the registration can be greatly compromised. On the other hand, the sCT generation is obtained from the trained model parameters, which may produce some fake structure inconsistent with the CBCT images. Therefore, this study implemented image registration based on MSnet and sCT generation based on CycleGAN to better improve the quality of CBCT images, and analyzed the effect of anatomical structure changes in pCT and CBCT scans on the accuracy of registration and sCT generation.

In this paper, we introduce the dataset acquisition and image processing in *Section 2.1*, deformable image registration and data preprocessing in *Section 2.2*, and the CycleGAN-based CBCT to sCT generation in *Section 2.3*. Then, we present the experimental results in *Section 3* and discuss the experimental results and related research in *Section 4*.

## Materials and Methods

### Dataset Acquisition and Image Processing

In this study, 115 cases of cervical cancer were retrospectively collected between June 2021 and October 2021 at Peking Union Medical College Hospital. The patients ranged in age from 32 to 73 years with a median age of 56 years. Among them, each patient includes 1–2 sets of pCT and the corresponding delineation information. The iCBCT was acquired when the patient underwent radiotherapy for the first time normally. Moreover, iCBCT could be obtained in each fraction when the radiotherapy was delivered in the Varian Halcyon 2.0 system. A total of 190 pairs of CT and first fraction iCBCT images were collected, of which 150 were used for model training, and 40 were used for model evaluation. The CT images were obtained on PHILIPS BrillianceTM Bigbore CT, which has a bore with a diameter of 85 cm. The plane resolution of the CT ranged from 0.962 mm × 0.962 mm to 1.365 mm × 1.365 mm, and the slice thickness was 5 mm. The iCBCT images were obtained from the Halcyon system, with a plane resolution ranging from 0.908 mm × 0.908 mm to 1.035 mm × 1.035 mm and a slice thickness of 2 mm. The range of iCBCT was mainly concentrated near the tumor target area, with a length of about 240 mm. Meanwhile, the scanning range of CT is longer than that of iCBCT and can completely cover the scanning range of iCBCT.

**Figure 1:**
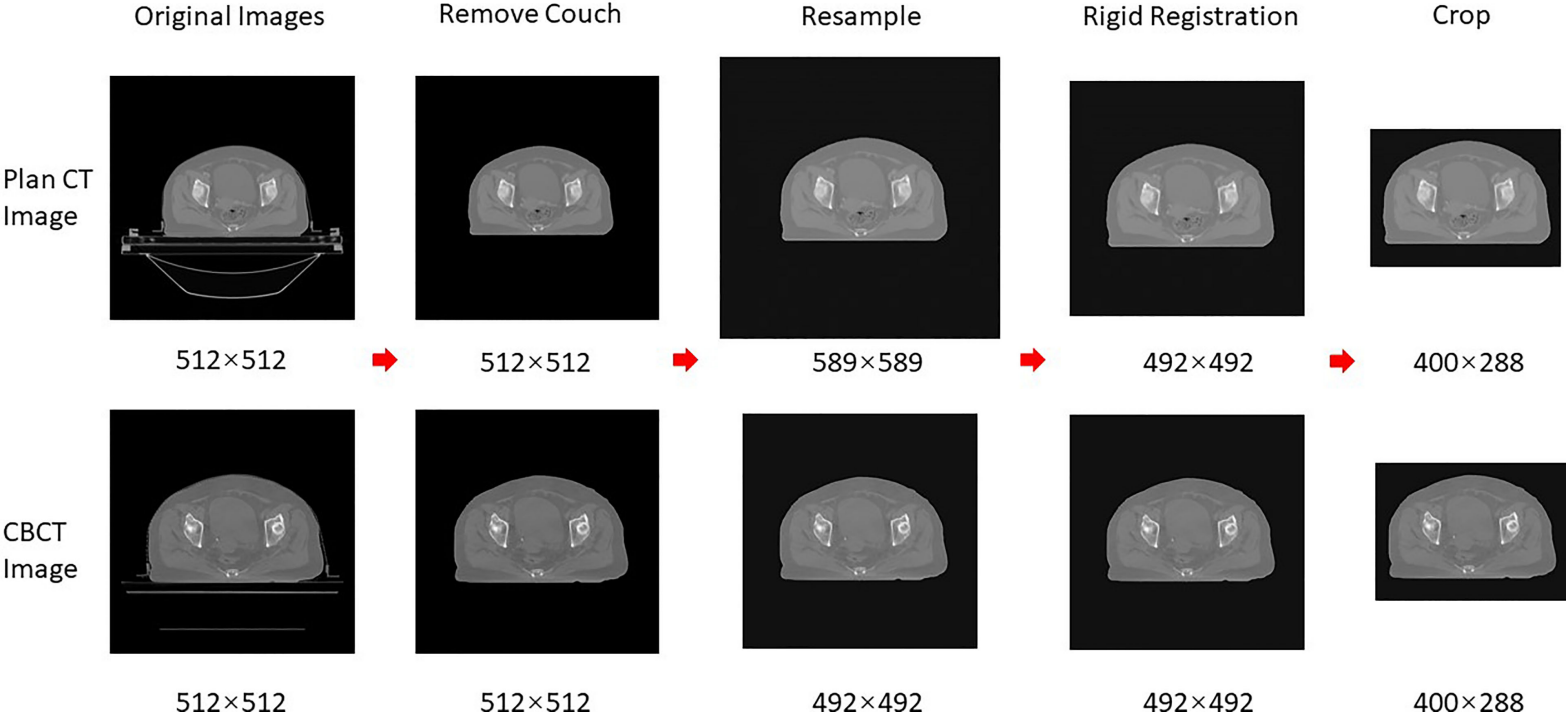
Schematic diagram of data preprocessing.

The data preprocessing was required before DIR and sCT generation. The common preprocessing is shown in **Figure 1**, which included removing couch, resampling, rigid alignment, and cropping; the specific preprocessing for registration and sCT generation will be introduced later. Firstly, the skin prediction model was combined with the image processing of expansion corrosion, which can quickly and accurately extract the skin mask. The interference of redundant information outside the body was removed, and the HU values outside the body were set to the HU value of the air (−1000). Secondly, the CBCT and CT images were resampled to 1 mm × 1 mm × 5 mm. Then, the CBCT images were set as fixed images, and the CT images were rigidly aligned to the CBCT images based on the ITK rigid registration method (25, 26). The redundant layers in the CT images were removed. Finally, the centroid of the skin mask was set as the image center; 400 × 288 voxels are cropped out of each layer of the image, which can completely contain the outline of the body. It should be emphasized that the entire image preprocessing is fully automatic without manual participation.

### Deformable Image Registration

Although common preprocessing was completed, additional data processing operations for registration required threshold cutoff and normalization. The threshold range of HU values is [−250, 200]; then, the pixel values of the image data were normalized and mapped to the range of (−1, 1).

**Figure 2:**
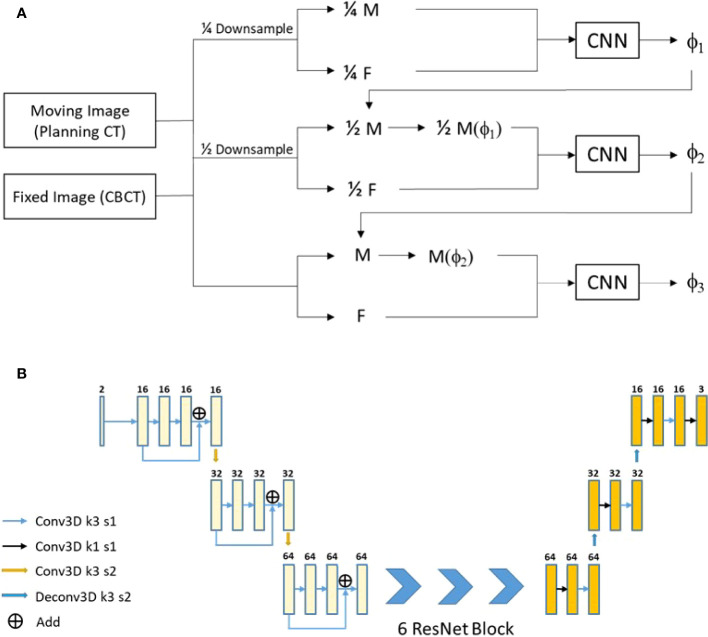
Our proposed registration method. **(A)** The network flow diagram. **(B)** The network architecture.

The used registration method was a 3D multi-stage cascade registration network, which was shown in **Figure 2** and realized the registration of pCT images to CBCT images. The network expected a pair of CT and CBCT images with 400 × 288 × 48 × 2 voxels and output a deformation field with 400 × 288 × 48 × 3 voxels. The network consists of three stages of registration, which achieved accurate registration from coarse to fine. The network architecture is shown in **Figure 2B**, which included two down-sampling layers and two up-sampling layers. Six ResNet Blocks (27) were used to increase the depth of the network and make the model easier to optimize. The loss function of the registration included the MIND (modality-independent neighborhood descriptor) loss (L_MIND_) (28, 29) and smoothing loss (L_smooth_) (30). The model was trained and tested on Nvidia Geforce RTX 3090. The batch was set to 20 with the model in stage 1, 4 in stage 2, and 1 in stage 3. The training required approximately 24 h for 200 epochs.

### CycleGAN-Based CBCT to sCT Generation

Additional data processing operations for sCT generation was required, which was according to the formula.


$$x=Tanh\left(\frac{x}{400}\right)$$

where the Tanh function was the hyperbolic tangent function, defined as


$$Tanh(x)=\frac{e^{x}-e^{-x}}{e^{x}+e^{-x}}$$

Because the final activation function of the generator model was Tanh, the CBCT and CT images were preprocessed by Tanh, which could improve the accuracy of sCT generation.

**Figure 3:**
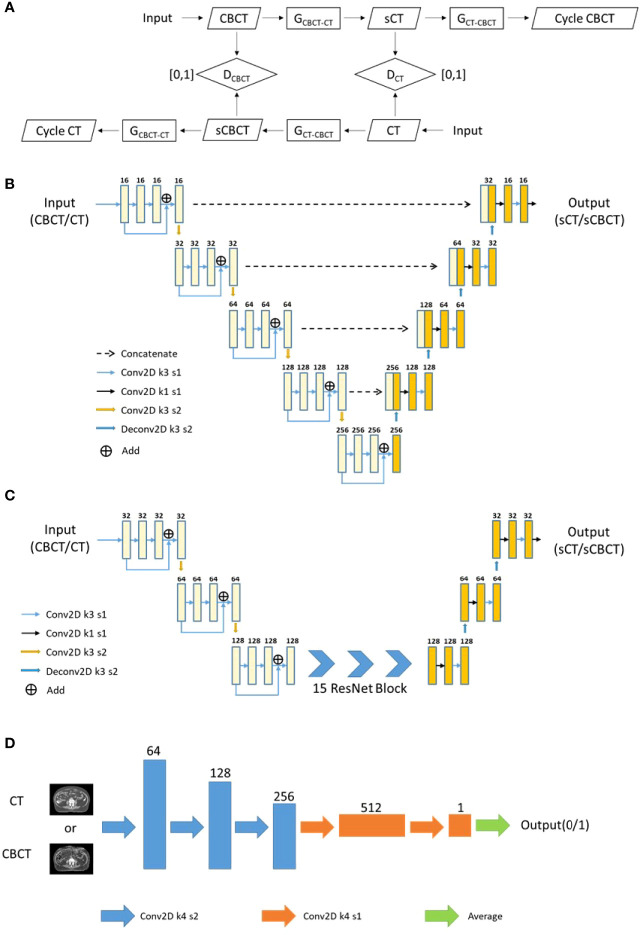
The flowchart and network architecture of sCT generation. **(A)** Architecture of CycleGAN. **(B)** U-net Generator. **(C)** Resnet Generator. **(D)** Discriminator.

The architecture of CycleGAN is shown in **Figure 3A**, which mainly included two generators (Gcbct-ct and Gct-cbct) and two discriminators (Dct and Dcbct): Gcbct-ct generated sCT from the CBCT image, Gct-cbct generated sCBCT from the CT image, Dct identified the sCT image from the real CT image, and Dcbct identified the sCBCT image from the real CBCT image. During the training process, Gcbct-ct would try to generate an sCT that made Dct indistinguishable as much as possible, and then Gct-cbct would convert the sCT image generated in the previous step into the CBCT image, called cycle CBCT, so as to make the CBCT image and the cycle CBCT image as consistent as possible. We compared the accuracy of different networks as generators, such as the U-net (**Figure 3B**) and Resnet (**Figure 3C**). The discriminators used the same architecture as shown in **Figure 3D**.

The loss function of the sCT generation consisted of three parts: ① Adversarial Loss *Ladv*, which could facilitate the distribution of the synthetic images similar to that of the images in the target. ② Cycle-consistency Loss *Lcycle*, which could serve as an indirect constraint of structure between the input and synthetic images. ③ Similarity-constraint Loss *Lsc*, which used the MIND loss to enforce the structural consistency between synthetic images and real images. LG is defined as follows and the hyperparameters *λ* and *μ* were set to 10.


LG=Ladν+λLcycle+μLsc

The model was trained and tested on Nvidia Geforce RTX 3090. Verified by extensive experiments, the batch was set to 6, the initial learning rate was set to 0.002, and the discrimination rate was set to 0.02. The epoch number was set to 200, and the learning rate decreased linearly from 0.002 to 0 in last 100 epochs.

### Deformable Image Registration Evaluation

In this study, 40 pairs of CBCT and CT images were used to evaluate the registration. Due to the poor quality of CBCT images, the distribution of HU values was also different from CT images; thus, the single-modal similarity measure was not accurate to evaluate the registration. Firstly, objective evaluation criteria were used for images, including normalized mutual information (NMI) and normalized cross-correlation (NCC). Then, the dice similarity coefficient (DSC) was used to evaluate the registration accuracy. The manual contours delineated on CBCT (Mask_CBCT) were used as the ground truth, the contours on the pCT image were propagated to the CBCT image (deformed mask, dMask) through the deformation matrix, and the DSC values of Mask_CBCT and dMask could reflect the accuracy of the registration. The organs for contour comparison included the bladder, spinal cord, femoral head left, femoral head right, and bone marrow.


(1) $$NMI(I_{1},I_{2})=2\frac{\sum_{i=1}^{I_{1}}\sum_{j=1}^{I_{2}}P(i,j)\log(\frac{P(i,j)}{P(i)P(j)})}{\left(-\sum_{i=1}^{I_{1}}P(i)\log(P(i)))+(-\sum_{j=1}^{I_{2}}P(j)\log(P(j))\right)}$$

(2) $$NCC(I_{1},I_{2})=\frac{1}{n_{i}n_{j}n_{k}}\sum_{x,y,z}^{n_{i}n_{j}n_{k}}\frac{\left(I_{1}(x,y,z)-\mu_{I_{1}})(I_{2}(x,y,z)-\mu_{I_{2}}\right)}{\left(\sigma_{I_{1}}\sigma_{I_{2}}\right)}$$

(3) $$DSC(V_{1},V_{2})=\frac{2(V_{1}\cap V_{2})}{V_{1}+V_{2}}$$

*I*_1_ and *I*_2_ represent two different images, *P*(*i*) means the probability distribution of the variable *i*, *I*(*x, y, z*) means the HU value of pixels (*x, y, z*) in image *I*. *n_i_n_j_n_k_* is the total number of pixels in image *I*. *µ* and *σ* represent the mean and the standard deviation of the HU value in an image. *V*_1_ and *V*_2_ represent the volume of the two contours for comparison, respectively

### Synthetic CT Image Quality Evaluation

The sCT evaluation criteria included mean absolute error (MAE), root mean square error (RMSE), peak signal-to-noise ratio (PSNR), and structural similarity (SSIM). The corresponding dpCT image with MSnet was used as the ground truth.


(4) $$MAE(I_{1},I_{2})=\frac{1}{n_{i}n_{j}n_{k}}\sum_{x,y,z}^{n_{i}n_{j}n_{k}}\left|I_{1}(x,y,z)-I_{2}(x,y,z)\right|$$

(5) $$RMSE(I_{1},I_{2})=\sqrt{\frac{1}{n_{i}n_{j}n_{k}}\sum_{x,y,z}^{n_{i}n_{j}n_{k}}{\left|I_{1}(x,y,z)-I_{2}(x,y,z)\right|}^{2}}$$

(6) $$PSNR(I_{1},I_{2})=10\times \log_{10}(\frac{MAX^{2}}{RMSE{\left(I_{1},I_{2}\right)}^{2}})$$

(7) $$SSIM(I_{1},I_{2})=\frac{\left(2\mu_{I_{1}}\mu_{I_{2}}+c_{1})(2\sigma_{I_{1},I_{2}}+c_{2}\right)}{\left(\mu_{I_{1}}2^{\;}+\mu_{I_{2}}2^{\;}+c_{1})(\sigma_{I_{1}}2^{\;}+\sigma_{I_{2}}2^{\;}+c_{2}\right)}$$

MAX was the maximum HU value in the selected image, and other parameters are similar to the above.

Considering the difference in the anatomical structure of the pCT and CBCT images, it is not complete to use the above evaluation criteria to evaluate sCT generation. The DSC was also used for sCT evaluation. The manual contours delineated on CBCT (Mask_CBCT) were regarded as the ground truth, and the physicians re-delineated the contours based on the generated sCT (Mask_sCT). The overlap between Mask_CBCT and Mask_sCT was calculated to evaluate the sCT accuracy. The organs for contour comparison included the bladder, spinal cord, femoral head left, femoral head right, and bone marrow.

## Results

### Deformable Image Registration

**Figure 4:**
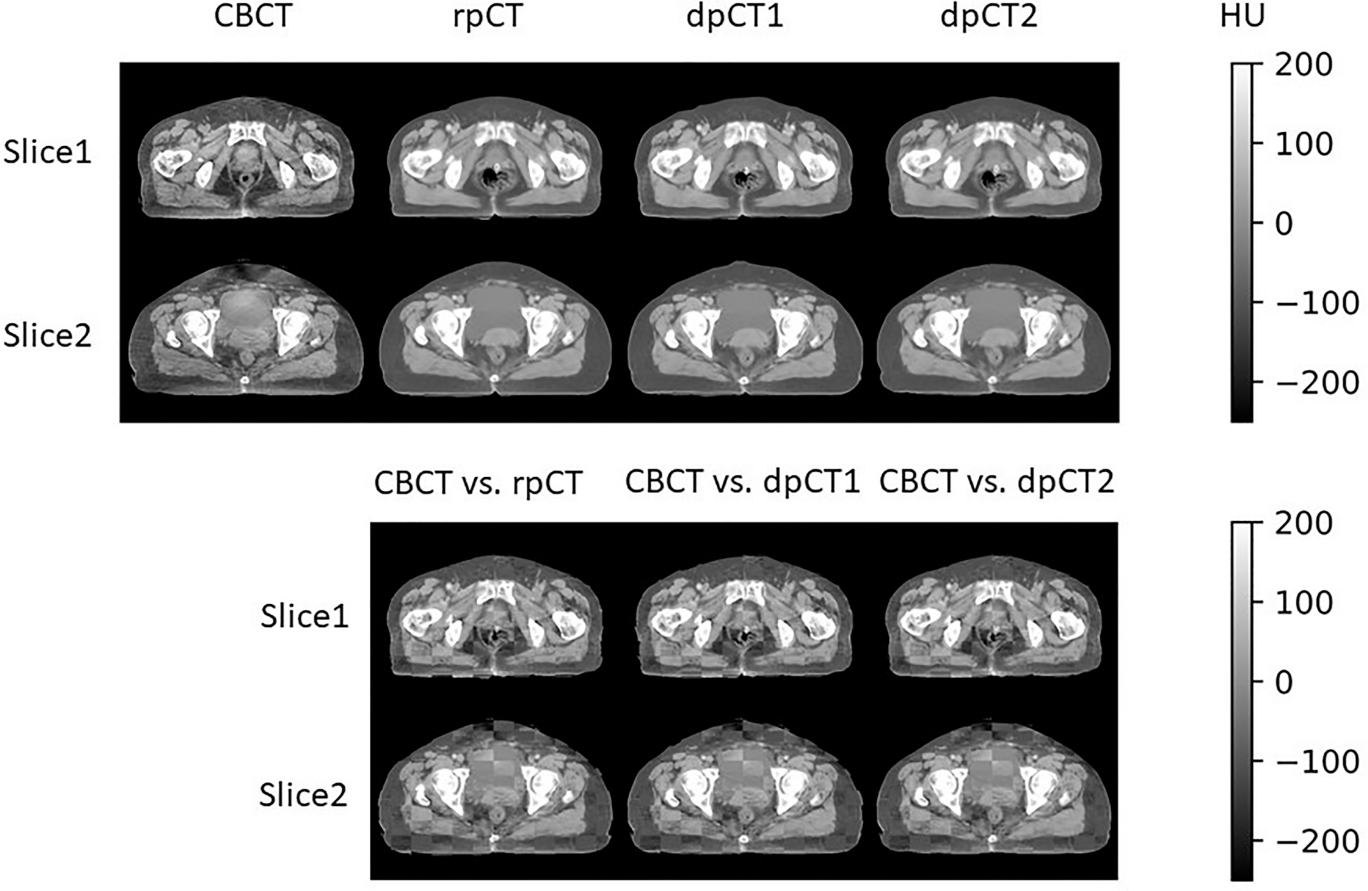
Visualization of registration result. rpCT, rigid planning CT; dpCT1, deformed planning CT with Elastix method; dpCT2, deformed planning CT with MSnet.

The DIR result of pCT and CBCT is shown in **Table 1**. Rigid registration was used for setup verification in the clinic and used for rigid alignment in our experiments, and we wanted to observe further improvement of DIR compared with rigid registration. MSnet registration was compared with the Elastix B-spline registration method (31, 32). It could be seen that both MSnet and the Elastix had improved the registration accuracy to some degree. In addition to the DSC of the bladder, MSnet was better than the Elastix in the evaluation of various indicators. **Figure 4** showed the difference between CT images and CBCT images before and after registration; MSnet had better skin contour alignment. In terms of time, it took 0.15 s for MSnet to get the dpCT for one case, while the Elastix method needed 30–50 s, about two hundred times faster.

**Table 1:** The registration result of pCT and CBCT (Ave ± Std).

		Rigid	Elastix	MSnet
NMI		0.350 ± 0.034	0.379 ± 0.033	0.397 ± 0.033
NCC		0.959 ± 0.009	0.969 ± 0.008	0.980 ± 0.005
DSC	Bladder	0.738 ± 0.120	0.769 ± 0.125	0.755 ± 0.121
	Spinal_Cord	0.631 ± 0.145	0.741 ± 0.075	0.765 ± 0.088
	Femoral_Head_L	0.882 ± 0.061	0.913 ± 0.022	0.918 ± 0.028
	Femoral_Head_R	0.878 ± 0.052	0.891 ± 0.142	0.916 ± 0.022
	Bone_Marrow	0.796 ± 0.071	0.858 ± 0.036	0.878 ± 0.031

### Synthetic CT Generation

**Figure 5:**
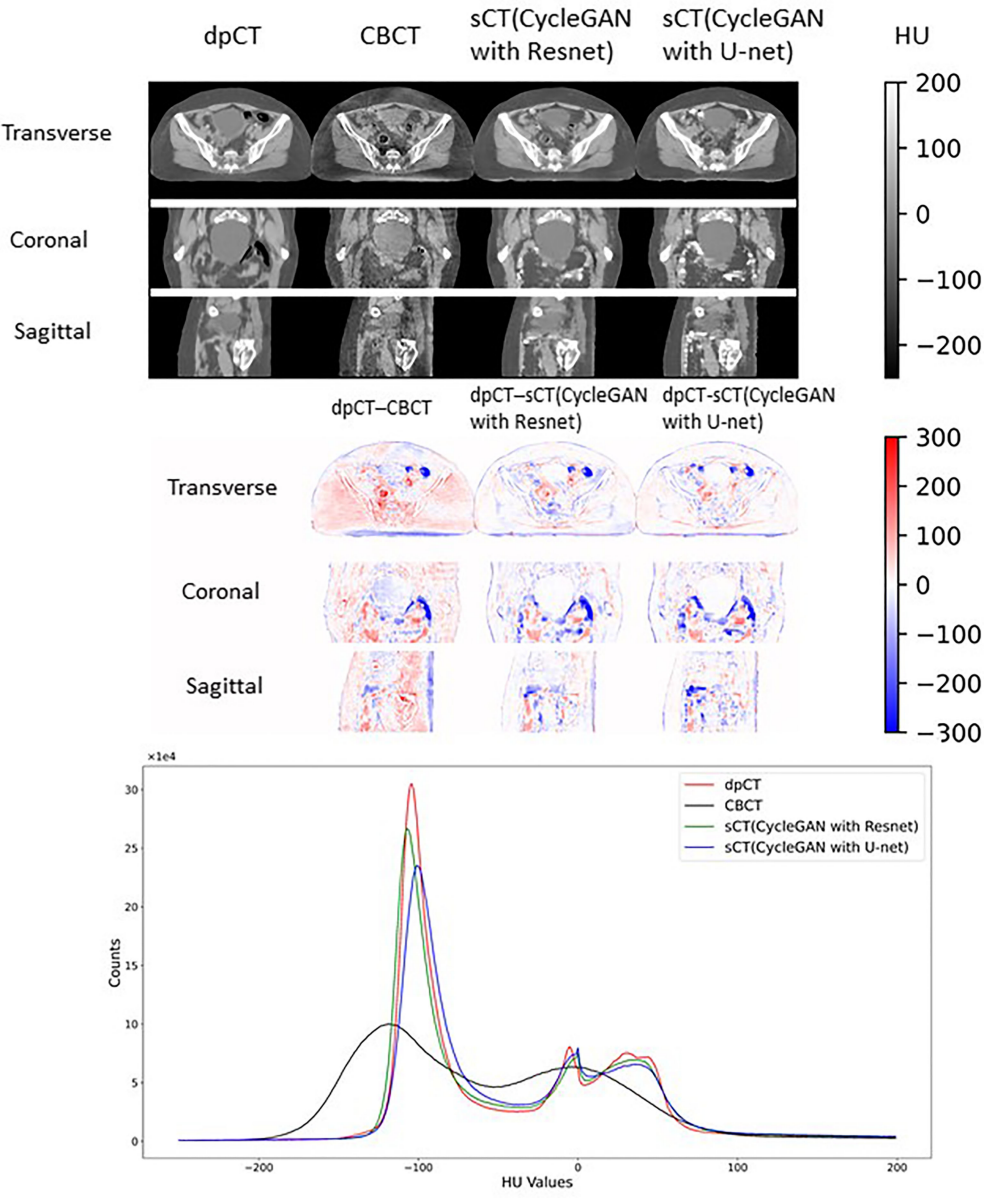
Visual comparison of dpCT, CBCT, sCT (CycleGAN with Resnet), and sCT (CycleGAN with U-net). The HU difference between two image sets. The HU histogram comparison of dpCT, CBCT, sCT (CycleGAN with Resnet), and sCT (CycleGAN with U-net).

**Figure 6:**
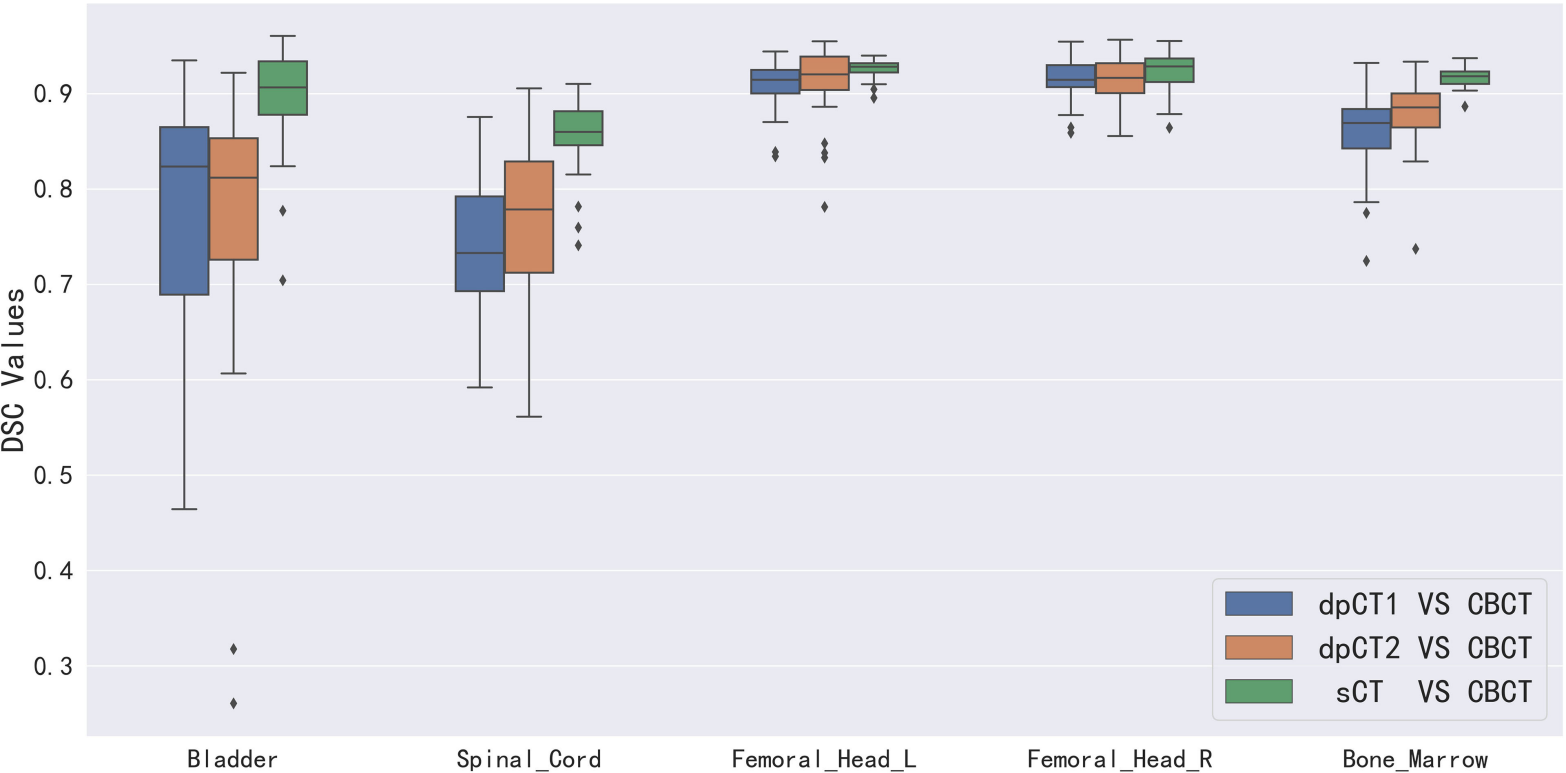
Boxplot of DSC values for registration and sCT generation. dpCT1, deformed planning CT with Elastix; dpCT2, deformed planning CT with MSnet.

**Table 2** shows the CBCT image quality improvement from CBCT images to sCT images, where CBCT images and sCT images were compared with dpCT by metrics including MAE, RMSE, PSNR, and SSIM. **Figure 5** showed visualization of sCT generation for one example. It can be seen from the results that the generator of Resnet with 15 ResNet blocks had a better effect than the generator of U-net with 5 down-sampling layers, which had significant improvement over CBCT in various indicators and less difference with real CT images. The results showed that the ResNet blocks could use feature combinations at different levels to improve CBCT image quality more accurately. Limited by the busy work and manpower, physicians only re-delineated the contours of organs on the sCT produced by the Resnet, which was compared with the contours of CBCT. The DSC results are shown in **Table 3**. It can be seen from the results that the accuracy of sCT was higher than the accuracy of registration. Except for the femoral head left and femoral head right, the remaining three organs had significant differences, which also showed that the sCT had higher structural consistency with CBCT images compared with dpCT. **Figure 6** showed the boxplot of DSC values for registration and sCT generation.

**Table 2:** The result of sCT generation (Ave ± Std).

	dpCT-CBCT	dpCT-sCT (Resnet)	dpCT-sCT (U-net)
MAE(HU)	51.23 ± 13.67	43.98 ± 10.74	46.71 ± 12.71
RMSE	121.09 ± 30.23	117.58 ± 28.22	127.96 ± 30.76
PSNR	20.01 ± 2.74	22.23 ± 2.61	20.00 ± 3.77
SSIM	0.623 ± 0.084	0.680 ± 0.050	0.685 ± 0.055

dpCT, deformed planning CT with MSnet.

**Table 3:** The comparison of registration and sCT (Ave ± Std).

	DSC (sCT, CBCT)	DSC (dpCT1, CBCT)	*p*1-values	DSC (dpCT2, CBCT)	*p*2-values
Bladder	0.884 ± 0.071	0.769 ± 0.125	*p* < 0.001	0.755 ± 0.121	<0.001
Spinal_Cord	0.850 ± 0.039	0.741 ± 0.075	*p* < 0.001	0.765 ± 0.088	<0.001
Femoral_Head_L	0.923 ± 0.010	0.913 ± 0.022	0.011	0.918 ± 0.028	0.265
Femoral_Head_R	0.921 ± 0.023	0.891 ± 0.142	0.217	0.916 ± 0.022	0.238
Bone_Marrow	0.916 ± 0.009	0.858 ± 0.036	*p* < 0.001	0.878 ± 0.031	<0.001

dpCT1, deformed planning CT with Elastix. p1-value, DSC (sCT, CBCT) vs. DSC (dpCT1, CBCT). dpCT2, deformed planning CT with MSnet. p2-value: DSC (sCT, CBCT) vs. DSC (dpCT2, CBCT).

**Figure 7:**
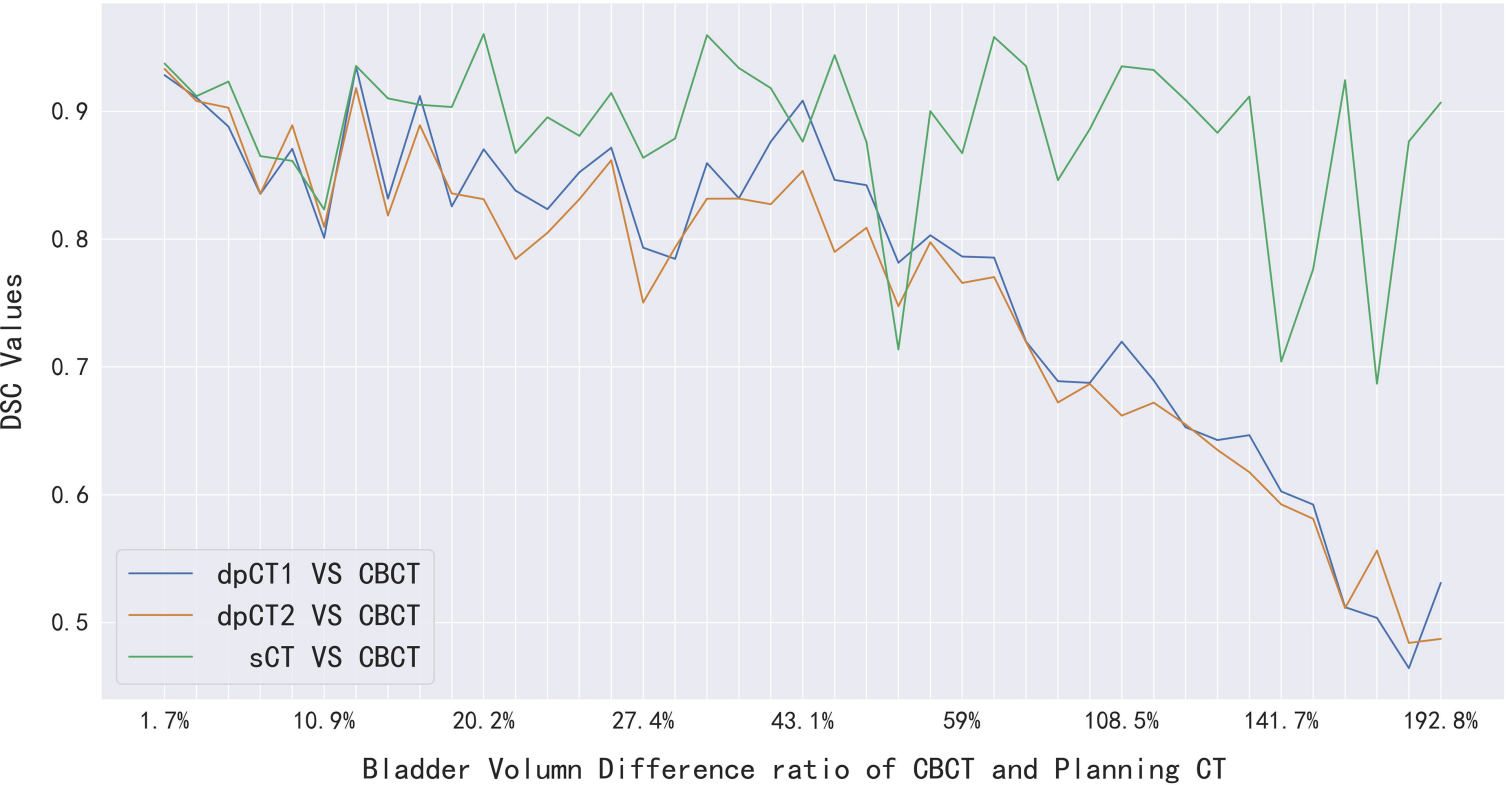
The effect of bladder volume difference on registration and sCT accuracy. dpCT1, deformed planning CT with Elastix; dpCT2, deformed planning CT with MSnet.

We analyzed the cases with poor registration performance, and found that these cases’ anatomical structures of pCT and CBCT were quite different, especially the bladder volume difference. When the volume difference was large, it was difficult to achieve good registration performance. Therefore, we calculated the volume difference of organs in pCT and CBCT (including the bladder, spinal cord, femoral head left, femoral head right, and bone marrow), and then statistically summarized the accuracy of registration and sCT with increasing volume difference. The results are shown in **Table 4**, in which it can be seen that the volume difference of bony structures (femoral head and pelvic) was small, most of the volume difference is less than 1%, and a small part may have a volume difference of less than 1% due to inconsistent delineation levels between the upper and lower ends. The main reason for the lower accuracy of the spinal cord was the different layers delineated in pCT and CBCT images. The bladder volume difference of pCT and CBCT was relatively large among the 40 cases in this study, only 9 had a volume difference of less than 20%, and 11 had a doubled volume difference (Diff > 100%). The DSC value of registration also changed from 0.874 to 0.587. The bladder volume difference was caused by the different degree of bladder filling during pCT scan and CBCT scan, which may be related to factors such as drinking water and waiting time. The above results showed that the volume difference had almost no effect on the accuracy of sCT, and had relatively little effect on the registration accuracy of bony structures (femoral head and pelvis). The volume difference had a great influence on the registration of soft tissues, especially the bladder in this study. **Figure 7** shows the effect of bladder volume difference on registration and sCT accuracy. With the increase of bladder volume difference, the delineation accuracy of the bladder in sCT was relatively stable, but the registration accuracy had dropped significantly.

**Table 4:** The effect of volume difference on registration and sCT accuracy (DSC: Ave ± Std).

	$Diff(V_{CBCT},V_{pCT})$	Counts	dpCT1	dpCT2	sCT
Bladder	<20%	9	0.874 ± 0.045	0.874 ± 0.043	0.898 ± 0.034
	20%–50%	13	0.846 ± 0.032	0.815 ± 0.029	0.905 ± 0.034
	50%–100%	7	0.750 ± 0.046	0.737 ± 0.427	0.858 ± 0.106
	>100%	11	0.596 ± 0.079	0.587 ± 0.066	0.859 ± 0.088
Spinal_Cord	<20%	13	0.763 ± 0.060	0.805 ± 0.079	0.854 ± 0.031
	20%–50%	18	0.768 ± 0.062	0.793 ± 0.041	0.854 ± 0.046
	50%–100%	5	0.680 ± 0.043	0.700 ± 0.026	0.848 ± 0.029
	>100%	4	0.620 ± 0.023	0.582 ± 0.244	0.814 ± 0.013
Femoral_Head_L	<1%	7	0.925 ± 0.012	0.931 ± 0.015	0.924 ± 0.007
	1%–3%	14	0.910 ± 0.027	0.902 ± 0.038	0.920 ± 0.011
	3%–5%	14	0.905 ± 0.018	0.925 ± 0.017	0.926 ± 0.009
	>5%	5	0.927 ± 0.010	0.924 ± 0.013	0.923 ± 0.006
Femoral_Head_R	<1%	6	0.911 ± 0.017	0.930 ± 0.018	0.926 ± 0.011
	1%–3%	10	0.917 ± 0.022	0.925 ± 0.018	0.926 ± 0.019
	3%–5%	14	0.915 ± 0.024	0.913 ± 0.022	0.912 ± 0.024
	>5%	10	0.910 ± 0.013	0.901 ± 0.021	0.922 ± 0.025
Bone_Marrow	<2%	6	0.863 ± 0.016	0.889 ± 0.019	0.920 ± 0.011
	2%–5%	17	0.871 ± 0.020	0.885 ± 0.019	0.917 ± 0.011
	5%–10%	11	0.857 ± 0.039	0.885 ± 0.018	0.917 ± 0.005
	>10%	6	0.818 ± 0.054	0.834 ± 0.047	0.909 ± 0.006

$Diff(V_{CBCT},V_{pCT})=\frac{\left|V_{CBCT}-V_{pCT}\right|}{MIN(V_{CBCT},V_{pCT})}\times \mathrm{100}\%$ dpCT1, deformed planning CT with Elastix; dpCT2, deformed planning CT with MSnet.

## Discussion

Due to the poor quality of CBCT images, which were often used for patient setup correction before radiotherapy in the current clinical practice, they cannot be used directly for accurate dose calculation. In this study, we had implemented two ways to improve the quality of CBCT images, including the registration of pCT to CBCT and the generation of sCT from CBCT. There existed many studies on CBCT-based dose calculations and CBCT-guided adaptive radiotherapy, which demonstrated that registration and sCT generation were acceptable within error tolerances (33–38). However, few studies had compared the accuracy difference of registration and sCT generation when the anatomical structure changes in pCT and CBCT scans. We conducted this study on cervical cancer cases; 150 pairs of CT and CBCT images were used for model training and 40 independent pairs were used to compare the accuracy. The manual contours delineated on CBCT images were regarded as the ground truth to evaluate the accuracy of registration and sCT generation.

For deformable image registration, we compared our proposed registration method (MSnet) with the Elastix B-spline method. MSnet achieved higher registration accuracy than the Elastix from the analysis of comprehensive indicators, and the time was significantly improved. It could be clearly seen from **Figure 4** that MSnet had higher accuracy in the alignment of skin and bony structures, and **Table 1** also presented the same result. If the bladder volume difference in CT and CBCT images was large, the registration could not be accurate. For the worst case, the DSC of bladder was less than 0.5, which might cause errors on dose calculation and be not eligible for precision radiotherapy. According to the AAPM TG 132 (39), the DSC of registration in the range 0.8–0.9 was acceptable. When the bladder volume difference was more than 50%, the registration was not satisfied.

CycleGAN was used to generate sCT from CBCT, which had aroused the interest of many researchers, including KV CBCT and MV CBCT. There are also related studies using different CNN structures as generator models. In this study, the U-net and Resnet were compared as generators to evaluate the accuracy of sCT; the Resnet achieved higher accuracy on our data for metrics such as MAE. Therefore, we generated sCT with the Resnet generator for the testing cases, and the physician re-delineated the contours on the sCT images. The results in **Table 3** show that the sCT accuracy was comparable with the registration on bony material, and the sCT had achieved obvious advantages in bladder and spinal cord. **Table 4** further illustrates that the volume difference had little effect on the delineation accuracy of the sCT, but gradually reduced the accuracy of the registration. When the anatomical structure greatly changes, the accuracy of the sCT is higher than that of the registration.

From the analysis of the above results, the sCT generated based on CBCT was superior to dpCT in terms of anatomical structure similarity with the CBCT structure. If the anatomical difference between pCT and CBCT was small, there was little difference between the two methods. Although sCT had higher accuracy, we thought that if the difference between the pCT and CBCT was small, the registration could better reflect the real structure of the case; after all, it was a real CT image. The sCT was generated by a series of parameters obtained from continuously optimizing the data in the training set, which may appear out of nothing compared with the CBCT image. For example, the cavity artifact in the CBCT image was very serious, and the information of the CBCT images was insufficient, which may bring errors in the post-processing correction. In addition, structures such as the bladder and the prostate were close to each other, and the HU values were also very similar, which cannot be identified on the sCT in some instance. Although some studies thought that this situation had little effect on the dose calculation [11], the errors did exist in anatomical structure.

We had studied two methods to improve the image quality of CBCT, and if the two methods could be effectively combined, they may lead to better clinical applications. Note that the difference in bladder volume between pCT images and CBCT images was a major factor affecting the registration accuracy, which could be used as a judgment condition for choosing two methods. We evaluated the accuracy of auto-segmentation on sCT, and the DSC of bladder was 0.874 ± 0.072, which can replace the contours on CBCT approximately. Firstly, we have the pCT images and corresponding contours. When the CBCT images were obtained before radiotherapy, the pCT was registered to the CBCT to obtain the propagated contours, especially the contours of the bladder (dpCT_bladder). Secondly, the CBCT was transformed to sCT, which can be used for auto-segmentation; we can get the contours of bladder on sCT (sCT_bladder). If the DSC of dpCT_bladder and sCT_bladder was above a certain threshold (e.g., DSC > 0.8), the dpCT and corresponding contours would be used. If it was below a certain threshold, the physician would check the auto-segmentation of the sCT for the current radiotherapy, and the generated sCT can be used for dose calculation and evaluation of adaptive radiotherapy. The above process can be done automatically in a short time (less than 1 min), which can be used for more accurate dose tracking.

Several limitations should be noted in this study. First, we selected five OARs to evaluate the accuracy of registration and sCT generation, but the target was the most important concern in clinical practice. It was difficult to delineate the target volume, the small intestine, and rectum on CBCT images due to the existence of artifacts, which were also controversial as the ground truth. In future work, the cases with small differences in anatomical structures can be selected to evaluate the accuracy of target delineation in the sCT images. Second, the focus of this study was to compare the accuracy of registration and sCT generation on structural similarity; the dosimetric differences would be done in our next work.

## Conclusion

We proposed two methods to improve the image quality of CBCT in this study. Both registration and sCT generation can effectively improve the image quality of CBCT. When the anatomical structure changes in pCT and CBCT scans were small, the accuracy of the registration and sCT was equivalent, and the anatomical structure of CBCT could be better represented by dpCT. When the anatomical structure changes were large, the accuracy of the sCT was higher than that of the registration, and the anatomical structure of CBCT could be better represented by sCT.

## Data Availability Statement

The raw data supporting the conclusions of this article will be made available by the authors, without undue reservation.

## Ethics Statement

The studies involving human participants were reviewed and approved by the Medical Ethics Committee of Peking Union Medical College Hospital. The patients/participants provided their written informed consent to participate in this study.

## Author Contributions

BY and YC conceived the experiments. YL and ZW collected the clinical dataset. XP, XX, and JQ designed the study and analyzed the result. BY, YC, YL, XP, and JQ participated in writing the manuscript. All authors contributed to the article and approved the submitted version.

## Funding

This work was supported by the China National Key R&D Program during the 13th Five-year Plan Period (Grant Nos. 2016YFC0105206 and 2016YFC0105207) and the University of Science and Technology of China (USTC) grants on “New Medicine Team Project: The ROADMAP Medical Physics Platform” (No. YD2140002002).

## Conflict of Interest

Author XP is employed by Anhui Wisdom Technology Co., Ltd.

The remaining authors declare that the research was conducted in the absence of any commercial or financial relationships that could be construed as a potential conflict of interest.

## Publisher’s Note

All claims expressed in this article are solely those of the authors and do not necessarily represent those of their affiliated organizations, or those of the publisher, the editors and the reviewers. Any product that may be evaluated in this article, or claim that may be made by its manufacturer, is not guaranteed or endorsed by the publisher.
