# Erratum: A comparison study between CNN-based deformed planning CT and CycleGAN-based synthetic CT methods for improving iCBCT image quality

**DOI:** 10.3389/fonc.2023.1159770

**Published:** 2023-03-07

**Authors:** Frontiers Production Office

**Affiliations:** Frontiers Media SA, Lausanne, Switzerland

**Keywords:** iCBCT, registration, sCT generation, pelvic, CycleGAN

Due to a production error, there was an error in affiliation 1. Instead of “Department of Radiation Oncology, Chinese Academy of Medical Sciences, Peking Union Medical College, Beijing, China”, it should be “Department of Radiation Oncology, Chinese Academy of Medical Sciences, Peking Union Medical College Hospital, Beijing, China”. The publisher apologizes for this mistake.

The original version of this article has been updated.

